# Could a behaviour change intervention be used to address under-recognition of work-related asthma in primary care? A systematic review

**DOI:** 10.3399/BJGPO.2024.0094

**Published:** 2025-06-04

**Authors:** Gareth Iestyn Walters, Harriet Foley, Christopher Charles Huntley, Anadil Naveed, Kimberley Nettleton, Christopher Reilly, Maximillian Thomas, Claire Walker, Kyrie Wheeler

**Affiliations:** 1 Institute of Applied Health, University of Birmingham, Birmingham, UK; 2 University Hospitals Birmingham NHS Foundation Trust, Birmingham, UK

**Keywords:** behaviour change, asthma, work-related asthma, asthma, occupational, primary health care

## Abstract

**Background:**

Work-related asthma (WRA) is prevalent yet under-recognised in UK primary care.

**Aim:**

To identify behaviour change interventions (BCIs) intended for use in primary care to identify WRA, or any other chronic disease (that could be adapted for use in WRA).

**Design & setting:**

A systematic review was conducted using narrative synthesis.

**Method:**

We searched Cochrane Central Register of Controlled Trials, Embase, PsycINFO, and Ovid MEDLINE databases (1946–2023) for studies describing development and/or evaluation of BCIs for case finding any chronic disease in primary care settings, aimed at either healthcare professionals and/or patients. Two blinded, independent reviewers screened abstracts and assessed full-text articles. We undertook narrative synthesis for outcomes of usability and effectiveness, and for BCI development processes.

**Results:**

We included 14 studies from 768 retrieved citations, comprising three randomised controlled trials, one uncontrolled experimental study, and 10 studies employing recognised multi-step behaviour change (BC) methodologies. None of the studies were concerned with identification of asthma. BCIs had been developed for facilitating screening programmes (five studies), implementing guidelines (three studies), and individual case finding (six studies). Five studies measured effectiveness, in terms of screening adherence rates, pre- or post-intervention competency, and satisfaction and usability, for clinicians, although none measured diagnostic rates.

**Conclusion:**

No single or multi-component BCIs have been developed specifically to aid identification of asthma or WRA, although other chronic diseases have been targeted. Development has used BC methodologies that involved gathering data from a range of sources, and developing content specific to defined at-risk populations, so are not immediately transferable. Such methodologies could be used similarly to develop a primary care-based BCI for WRA.

## How this fits in

Work-related asthma (WRA) is poorly recognised in primary care, with significant delays in diagnosis that lead to poor health and employment outcomes for affected individuals. Whether behavioural science can aid changing the practice of healthcare professionals or behaviour of such patients is unknown. This systematic review identified no studies that describe the development and/or effectiveness of a behaviour change intervention (BCI) to aid identification of WRA in primary care. However, a variety of BCIs have been developed specifically to identify other chronic diseases; these are unlikely to be immediately transferable to WRA, but this approach to intervention development could be taken to design and evaluate such an intervention for identifying WRA in primary care settings.

## Introduction

WRA includes asthma caused by exposures at work (occupational asthma) and existing asthma worsened by conditions at work (work-exacerbated asthma). The impetus for this study derives from our clinical experience of WRA as a markedly under-recognised chronic condition. If diagnosis is delayed or missed, affected individuals may suffer poor outcomes in terms of respiratory health and employment,^1,2^ and the societal impacts of healthcare-related costs and productivity losses are significant, with desktop estimates of social cost for the UK in the region of £1 billion per decade.^3^ Existing guidelines recommend that attending healthcare professionals (HCP) ask adult patients with new, reactivated, or worsening asthma symptoms about the nature of their job, about any relationship of their asthma symptoms with work, and seek expert advice when a relationship is demonstrated.^1,4^ However, in the UK and elsewhere, it is evident that these guidelines and their previous iterations have not been implemented successfully in a variety of settings, including primary care.^5–7^ In primary care, barriers to diagnosis for patients include poor understanding of asthma and work context, fear of job security and economic loss, and the assumption of insolubility.^8,9^ For primary care, barriers for HCPs include inadequate training and clinical experience, beliefs and perceptions about disease occurrence, pressures on specialist referral, lack of continuity of care, and time constraints.^10^

BCIs, including education and training aimed at modifying clinical behaviours, audit and feedback, or enablement of collaborative team-based approaches, are broadly effective in changing the individual or collective practice of primary care HCPs.^11^ Additionally, similar approaches may be engaged to influence the health-seeking behaviour of patients. A simple or complex BCI intended to increase early identification of WRA in primary care could be developed and evaluated for clinical effectiveness. An important step, and the rationale for this review, would be to see whether such interventions already exist, or whether similar initiatives from other chronic disease areas could be adapted for this purpose.

### Review questions

1. Have BCIs, aimed at modifying health-seeking or clinical behaviour, been developed and/or evaluated for identifying 1) WRA; or 2) any chronic disease in primary care settings?
2. Have any evaluations of effectiveness of these BCIs, in aiding identification of such chronic diseases, been performed?

## Method

This systematic review has been reported according to the Preferred Reporting Items for Systematic reviews and Meta-Analyses (PRISMA) updated statement and abstract checklist.^12^ The protocol was registered on the international PROPSERO database of systematic reviews on 19 April 2023.

### Literature search

The literature search was undertaken during the week starting 24 April 2023, via institutional access to the Ovid interface, and included the following databases: Cochrane Central Register of Controlled Trials (to 6 March 2023), Embase (1974–6 March 2023), Ovid MEDLINE (1946–6 March 2023), and the American Psychological Association’s PsycINFO (1967–6 March 2023). Search terms combined synonyms for the following three elements: BCIs; diagnosis; and primary care. The search strategy is shown in Supplementary Table S1. Reference lists from studies meeting the inclusion criteria were searched to identify any missed relevant studies.

### Inclusion criteria

Any study examining either the development, effectiveness, or both, of the BCI(s) in question, to include qualitative, observational, or experimental studies in biomedical and social science journals. These may have included case reports, case series, or published conference abstracts.

### Exclusion criteria

1. Books, book chapters, theses, dissertations, systematic or narrative reviews, opinion pieces, and protocols (unless a protocol for an effectiveness trial also describes the development of the intervention);
2. abstracts not in English; and
3. non-human research (for example, laboratory studies).

### Condition being studied

Any chronic disease, defined here as a disease of any bodily system likely to remain active or require treatment for >1 year, and that affects day-to-day functioning, requires medical treatment, or negatively impacts life expectancy. Common examples include hypertension, diabetes mellitus, epilepsy, depression, multiple sclerosis, and coeliac disease (not exhaustive). This definition also includes solid-body and haematological cancers.

### Populations being studied

1. Studies undertaken in any primary care population, with no geographical restriction; and
2. studies in which the target groups are either patients or primary care HCPs, or both.

### Interventions being studied

Any tool, intervention, or initiative described as a ‘behaviour change intervention’ or in such terms, designed to change the health-seeking behaviour of patients or the clinical behaviour of primary care HCPs, to aid diagnosis of any chronic disease. This did not include BCIs aimed at uptake of lifestyle modifications or treatments to prevent onset of disease (primary prevention and health promotion). BCIs aimed at increasing participation in screening programmes were considered for inclusion, although studies that described development or evaluation of screening programmes *per se* were excluded from this review.

### Comparator groups being studied

For some relevant and included study designs, no comparator group will have been evident (for example, studies describing the development of BCIs may have been mixed-methods, qualitative, protocols for subsequent trials, or case series). Studies evaluating the effectiveness of BCIs may have included randomised controlled trials (RCTs), where the comparator groups were either existing standards of care or head-to-head comparisons between novel interventions; or may have been uncontrolled trials.

### Primary outcomes

1. Related to development of BCI: theoretical framework used (if any), method(s) of development, construct, function of BCI, and any validation undertaken.
2. Related to clinical effectiveness of BCI: any effect measure (for example, risk ratios, prevalence ratios, and incidence rate ratios) depending on the experimental study design.

### Data selection

The results of the literature search were exported in to the Rayyan web-based application for abstract screening and automatically de-duplicated. Each abstract was screened independently and blinded by two reviewers, and any abstract included by at least one reviewer was shortlisted for full-text review. Full-text review was carried out independently and blinded by two reviewers, and a third independent review and discussion with consensus between reviewers undertaken where disagreement occurred. Primary reasons for exclusion were documented according to the categories shown in Supplementary Table S2.

### Data gathering

The following data were gathered by the primary author according to a standardised template (detailed in Supplementary Table S3): author(s); year; region; rationale; study design; population; disease entity; BCI; theoretical framework used; construct and function of BCI; and any validation undertaken. For RCTs, comparator groups, effect measures, and statistical significance were also collected. The data from a small sample (>10%, three studies) were checked for accuracy by a reviewer.

### Risk of bias and quality assessment

For each included full-text article, the primary author and a second reviewer undertook blinded quality assessments (see Supplementary Tables S4–S6). Where the researchers had employed a conventional study design as the primary methodology (that is, RCT or uncontrolled trial), a standardised Joanna Briggs Institute (JBI) critical appraisal tool was used to assess quality and risk of bias.^13–15^ These were chosen in order to assess quality and risk of bias at study and outcome levels, for a variety of study designs. Where methodology was non-traditional (that is, a multi-step or multi-component BCI methodology) then a narrative appraisal was undertaken, and any factors that would reduce confidence in the study design documented. Any disagreements were moderated by consensus.

### Data synthesis

Included studies were grouped primarily by the intended scope of the developed BCI (that is, facilitation of screening, uptake of existing guidelines, and individual case finding) and summary of overall study design provided in terms of geographical reach, aim (BCI development, evaluation, or both), target group (patients, HCPs, or both), and disease area. No quantitative synthesis or meta-analysis was planned, since pilot searches had revealed heterogeneous study designs, target populations, and high likelihood of multi-component studies being included. Studies were considered to have BC methodology if multi-step (>1) components were included to develop or refine BCIs; any underlying theoretical framework was assessed.

Data were tabulated where possible, grouped by variables, that is, study design (for example, BC methodology and experimental), disease, target group (patients and/or HCPs), narrative description of function, and construct. Effectiveness studies were grouped by summaries of intervention and comparator groups, main outcomes, and effect sizes with significance and confidence intervals. An accompanying narrative synthesis of quantitative data was undertaken. It was also intended to make recommendations for use of existing BCIs in the context of WRA.

## Results

**Figure 1.**
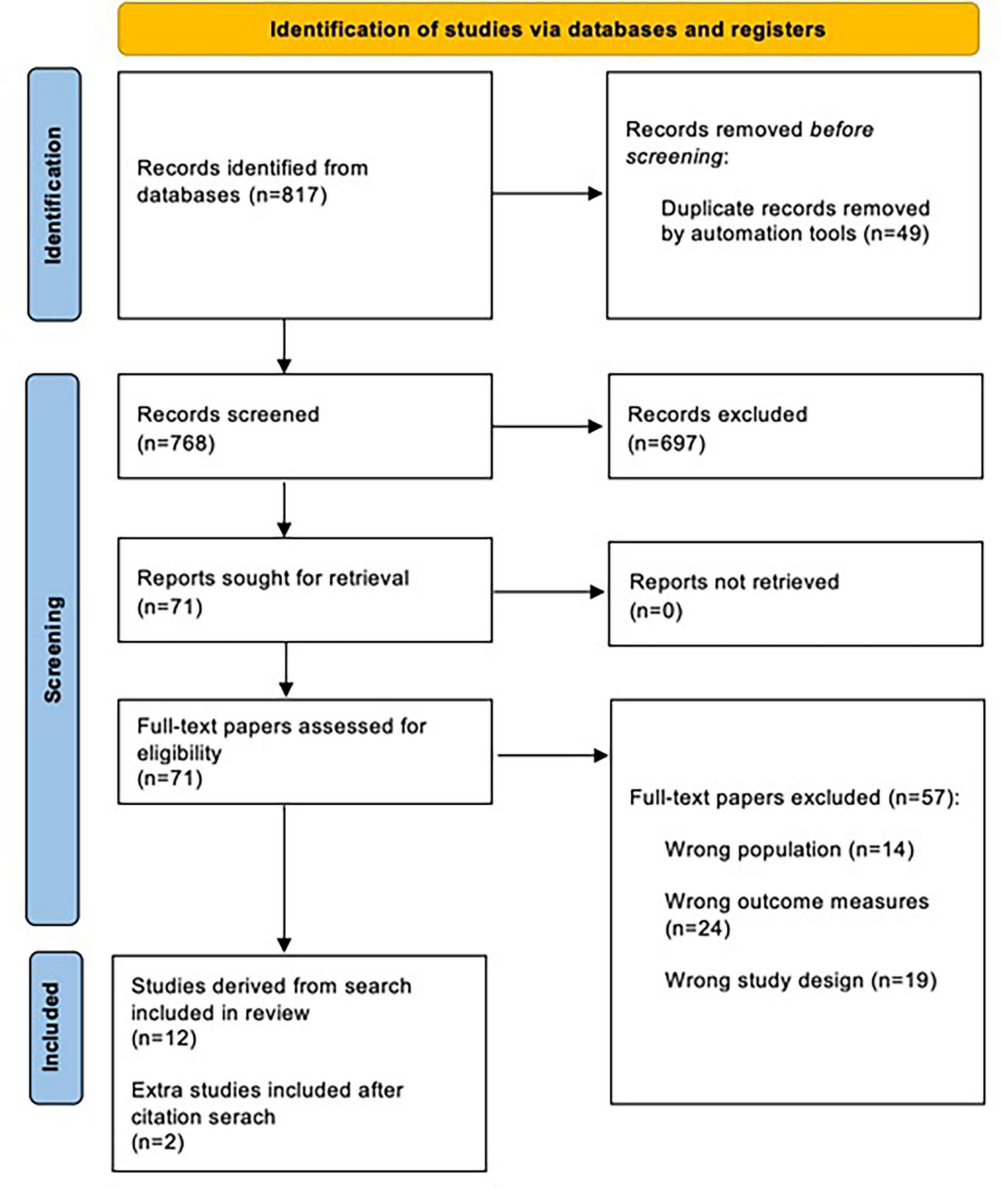
Preferred Reporting Items for Systematic reviews and Meta-Analyses (PRISMA) study flowchart^12^

From the initial database literature searches, 768 discrete citations were included for abstract screening following automated software de-duplication (see PRISMA flowchart in Figure 1). Seventy-one articles (9%) were shortlisted by at least one abstract reviewer and included for full-text review. On full-text review, agreement was found between both reviewers in 55/71 articles (77%; including 16 for inclusion and 39 for exclusion), with no further studies added after third review and moderation of disagreement; two additional articles identified through screening of reference lists by the lead author were included. Thus, 18 articles were initially included in the subsequent evidence synthesis, comprising 14 full studies and four conference abstracts. Reasons for exclusion at full-text review stage were as follows: wrong outcome (*n* = 24; that is, not BCI development or evaluation of effectiveness), wrong population (*n* = 14), and wrong study design (*n* = 19; for example, protocols, reviews, and opinion pieces). Four further articles (three full studies and one conference abstract) describing qualitative methodologies that were initially included were subsequently excluded during data synthesis, as the research team felt that solely qualitative studies would not constitute BC methodology as per the definition outlined in the method seection. Thus, the final number of included articles was 14.^16–29^ A list of the excluded studies at full-text review is available from the authors on request. Where conference abstracts have been included, a search for a subsequent full study has been undertaken, and none of these had been peer-reviewed and published at the time of writing.

### Quality and risk of bias assessments

Quality and risk of bias assessments are shown in Supplementary Tables S4–S6. There were three RCTs,^16,28,29^ one quasi-RCT,^17^ and 10 studies describing BCI development using BC methodology.^18–27^ Included RCTs lacked methodological detail on selection and allocation of participants or sites, in particular the process of randomisation, and on blinding of those evaluating outcomes to allocation; one of these articles^16^ was a conference abstract, so many methodological details were absent. The two full-article RCTs were published in subject-specific journals, so may have lacked the rigorous methodological detail required by a clinical trials journal.^28,29^ The trial with pre- and post-intervention outcome measurements, authored by Porcheret *et al,* ^17^ was nested in a larger RCT described elsewhere, but recruitment and dropout are described in detail.

Critical appraisal of 10 studies employing multi-step BC methodologies revealed inter-study variation, with no single standardised methodology or framework employed.^18–27^ Most commonly the theoretical domains framework (TDF) was used to establish barriers and enablers, with many of these studies applying the behaviour change wheel (BCW) to map barriers and enablers to intervention functions, behaviour change techniques (BCT) and intervention components, and APEASE (acceptability, practicability, effectiveness, affordability, side-effects [or safety], and equity) criteria for final selection.^18–25^ Two studies employed the Medical Research Council (MRC) complex interventions framework.^26,27,30,31^ There were methodological differences between studies for establishing barriers and enablers, identifying and selecting BCTs, and no standardised method for reporting multi-step methods; indeed, owing to the need to report multiple work packages in BC methodology studies, the qualitative elements, for example, lacked the usual reporting rigour of peer-reviewed and published qualitative research. In some BC methodology articles, individual work packages had been described in detail in separate citations, signposted from the article.^20,21,28^

### Narrative synthesis

Study characteristics for all included articles are shown in Supplementary Table S7, grouped by scope of BCI, and displayed with primary methodology, study population, intervention group, comparators, and relevant outcomes.

#### Facilitation of screening programmes

Five studies evaluated effectiveness of BCIs in facilitating a screening programme.^16,18,23,28,29^ Rubenstein *et al* ^29^ undertook a practice cluster RCT of an existing patient-facing internet-based risk assessment tool for stratifying personal risk and providing tailored messages. Absolute screening adherence increased in both intervention and control groups (standard care) at all levels of personal risk, although there were no significant differences between the two groups (unadjusted or adjusted for risk); the authors noted high baseline screening rates in the sample population. Kronish *et al* ^16^ developed a multi-component BCI (clinician, nursing, and patient education and information) for hypertension screening using BC methodology, which was then evaluated by practice cluster RCT against standard care. There was a significant relative increase in ordering and completion of ambulatory and home blood pressure monitoring in the intervention group over the control practices: 0.5% to 4.0%, *P*<0.001 versus 3.1% to 2.8%, *P* = 0.66 for ordering, and 0.5% to 3.0%, *P*<0.001 versus 2.2% to 2.0%, *P* = 0.76 for completion, although absolute adherence levels remained low. Larkey *et al* ^28^ undertook an RCT of BCIs to encourage colorectal cancer screening adherence in low income and minority ethnic groups. Head-to-head comparison of ‘storytelling’ (a video created from personal stories composited into a drama) versus personal risk control (a risk assessment tool based on the Harvard Cancer Risk Index) found no significant differences in screening adherence (37% and 42% screened, respectively).

Two studies described development of BCIs using methodologies underpinned by the TDF, aimed at increasing screening adherence; these were for cervical cancer,^18^ comprising a patient leaflet and video animation for HCP training, and diabetic retinopathy,^23^ incorporating a range of BC techniques (audit and feedback, electronic prompts targeting HCPs, HCP-endorsed reminders [face-to-face, telephone, and letter], and patient leaflet). Riordan *et al* ^23^ employed a multi-step BCW methodology and measured acceptability and feasibility of their BC techniques via stakeholder consensus groups, refining the final BCI by applying APEASE criteria.^32^

#### Implementation of diagnostic guidelines

Three studies employed multi-step BC methodology.^17,21,22^ Moise *et al* ^21^ developed and refined a multi-component BCI aimed at increasing uptake of hypertension guidelines by patients and HCPs, comprising exploration of barriers via qualitative focus groups, mapping of barriers to functions using the BCW, and feasibility evaluation through stakeholder interviews. Porcheret *et al* ^22^ drew on four theoretical frameworks: implementation of change model, TDF, theoretical mapping of behavioural determinants to BCTs (after Michie *et al^33^*), and principles of adult learning, in order to develop an enhanced consultation for identifying osteoarthritis through implementation of UK national guidelines. They developed and refined workshops for general practice that incorporated BCIs developed via 1) HCP consensus discussion; 2) GP focus groups; and 3) review of known enablers and barriers to innovation implementation; 10 BC techniques were incorporated including information provision, skills rehearsal, and persuasive communication. A subsequent effectiveness study by the same group^17^ described an uncontrolled trial of the final workshop, with GP competency measured through review of a video-recorded simulated consultation, before and after the intervention; competency score increased significantly at two time points, 1 month (*P* = 0.001) and 5 months (*P* = 0.001) after the workshop.

#### Individual case finding

All articles within this scope (*n* = 6) used multi-step BC methodologies and describe development of BCIs to aid individual case finding in: unspecified long-terms conditions,^19^ osteoporosis,^24^ first episode psychosis,^26^ lung cancer,^27^ unspecified cancers,^20^ and chronic kidney disease^25^ (see Supplementary Table S8). Four studies used multi-step BCW frameworks. Jinks *et al* ^19^ developed their multi-component enhanced consultation, which included tools for HCPs for case finding (not described in any further detail in conference abstract) using the TDF and COM-B framework, with steps comprising evidence synthesis, community of practice via stakeholder workshops, and thematic analysis of focus groups to assess training needs. Smits *et al* ^20^ also employed the TDF and BCW framework to refine content and delivery of an existing BCI, ‘The health check’, a touchscreen questionnaire, which is delivered face-to-face by a trained lay adviser. It aims to raise awareness of cancer risk and encourage health-seeking behaviour in the presence of symptoms, and specifically targets patients aged ≥40 years living in socioeconomically deprived communities. The authors also applied APEASE criteria to make judgements on the most appropriate BCI functions for their objectives. Tuot *et al* ^25^ developed a patient-facing online risk calculator (brief questionnaire, risk assessment, and self-management tools) and a clinician-facing ‘Clinical Practice Toolkit’ platform for identifying chronic kidney disease in at-risk populations. The clinician toolkit included a population-risk identifier, education about detection and management, patient health literacy assessment, and quality improvement intervention suggestions. The authors used a TDF and BCW framework in a multi-step process, which comprised establishing barriers and enablers to diagnosis (for example, awareness of guidelines and availability of data analytics) from qualitative data, and mapping these to intervention functions (in this case: education, persuasion, enablement, and modelling) to develop the BCI. Toh *et al* ^24^ have used thematic analysis of primary care stakeholder interviews (patients, pharmacists, nurses, and doctors), subsequently applying the BCW to identify a multi-component BCI for osteoporosis case finding consisting of: 1) a pharmacist-led risk assessment for osteoporosis; 2) an education session; and 3) restructuring of the current practice that incorporates this intervention into daily clinic practice.

Two studies employed the MRC framework for complex interventions; Lester *et al* ^26^ developed an educational BCI for GPs consulting on first-episode psychosis, via a multi-step process, including a literature review, analysis of data from focus groups, and feedback gained from surveys of GP users following initial and booster training. The BCI was well received among GPs, with an increase in self-reported knowledge of, skills within, and attitudes toward psychotic illness. Smith *et al* ^27^ used the MRC framework to develop a complex intervention for symptomatic patients at risk of lung cancer, which comprised a detailed self-help manual and extended consultation with a trained research nurse, at which specific action plans were devised. Elements comprised evidence review, multidisciplinary group design, and thematic analysis of user focus group data.

## Discussion

### Summary

This review identified no study describing development of single or multi-component BCIs for the identification of asthma or WRA specifically. More generally BCI development has focused on adherence to screening programmes, implementation of guidelines, and improving individual case finding (for example, through performance feedback, risk assessment, and medical education). The majority of studies have drawn on qualitative research with patients and/or HCPs to define behaviours that require change, usually resulting in identification of multiple BCTs and therefore multi-component BCIs. Additionally, a number of theoretical frameworks have been employed, resulting in multi-step development processes, most commonly the TDF (±BCW), but also implementation of change theory and the MRC complex interventions framework. Evaluation of BCIs for effectiveness in implementation of guidelines and individual case finding had been limited to outcomes related to diagnostic confidence, competency, improvement in learning, and other self-reported measures among HCPs; three RCTs of BCIs, which aimed to increase screening programme uptake, have given both positive and negative results, depending on background levels of absolute risk.

### Strengths and limitations

We have aligned our methods and reporting with the standardised PRISMA checklist and used established quality and risk of bias assessment tools as far as possible. However, no tool or benchmark for assessment of multi-step BC methodologies was available at the time of writing. Often owing to the number of work packages involved in a BC methodology study, detail was missing on methods and conduct of individual components, seen most frequently with qualitative methods, although occasionally individual work packages had been published separately. Literature searches were undertaken using search terms that accounted for various spellings of behaviour and acronyms for behaviour change intervention. However, it is possible that such interventions have been missed owing to being labelled otherwise.

### Comparison with existing literature

**Figure 2.**
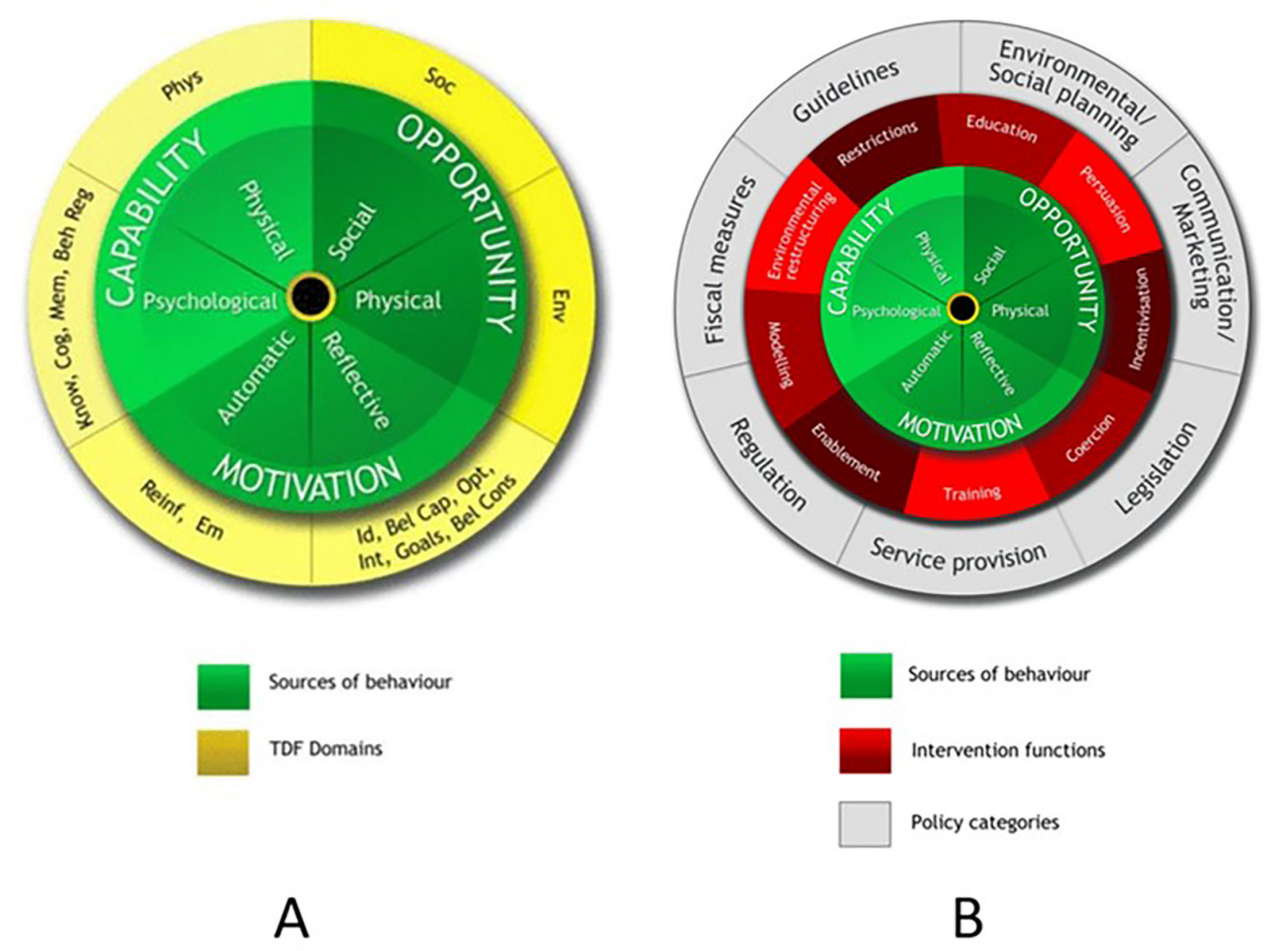
The behaviour change wheel (BCW): a new method for characterising and designing behaviour change interventions. A) Mapping between theoretical domains framework (TDF) and the COM-B model, frameworks for understanding behaviour and analysing relevant qualitative data; and B) the BCW. Figure 2A has been reproduced from Atkins *et al* ^39^ under Creative Commons Attribution 4.0 International Licence (http://creativecommons.org/licenses/by/4.0/). Figure 2B has been reproduced from Michie *et al* ^34^ under Creative Commons Attribution 2.0 Licence (http://creativecommons.org/licenses/by/2.0). Beh Reg = behavioural regulation. Bel Cap = beliefs about capabilities. Bel Cons = beliefs about consequences. Cog = cognitive and interpersonal skills. Em = emotion. Env = environmental context and resources. Id = social or professional role and identity. Int = intentions. Know = knowledge. Mem = memory, attention, and decision processes. Opt = optimism. Phys = physical skills. Reinf = reinforcement. Soc = social influences. TDF = theoretical domains framework.

The most encountered framework for research was the TDF, where qualitative methods have been employed to gather data to establish barriers and enablers of behaviour change. This has been frequently undertaken in combination with the more recently developed and simpler COM-B model of behaviour (capability, opportunity, and motivation are the key ingredients of behaviour change), and the BCW approach to intervention design, with demonstrable steps to identification of intervention functions and a range of suitable BCTs. Following a systematic literature review of intervention frameworks, Michie *et al* ^34^ developed the BCW framework given that no existing framework (including the MRC complex interventions framework) met their criteria of comprehensive coverage of intervention or policy function, coherence, and link to an overarching model of behaviour, including the MRC complex intervention framework. Indeed, interventions are commonly designed without consideration of the type of intervention likely to be effective, rationale for choice of interventions, the full range of possible influences on behaviour, and understanding the target behaviour.^35,36^ Many frameworks exist but have not been intended for or been tested in healthcare settings. The BCW framework (Figure 2) consists of the COM-B model at the core with its three critical components; surrounding this are nine intervention functions and seven policy categories that aim to address any COM-B components identified as deficient. Thus, each domain of TDF or COM-B can be used with the BCW to identify BCTs and design and refine interventions (Figure 2).

The eight-step process outlined by Michie *et al* ^34^ standardises a comprehensive approach to intervention development as follows: 1) define the problem in behavioural terms; 2) select the target behaviour(s) most likely to bring about change to address the problem; 3) specify the target behaviour in as much detail as possible; 4) identify what needs to shift in order to achieve the target behaviour; 5) identify intervention functions; 6) identify policy categories; 7) identify behavioural change techniques; and 8) identify mode of delivery. In a further step, APEASE criteria^32^ can be used to refine or select BCTs and their delivery, for an intervention, by considering a wider context of affordability, practicability, cost-effectiveness, acceptability, safety, and equity; in three included studies^20,21,23^ this was undertaken via consensus methods and evidence review. Although now popular in healthcare research (and elsewhere) for its comprehensive and practical approach to intervention design, limitations have been acknowledged, in terms of over-simplifying human behaviour, lack of stakeholder involvement including the target population, vagueness where judgement is required for decision making, and the requirement to understand psychological processes.^37^

### Implications for research and practice

The first aim of this study was to identify existing BCIs for use in primary care settings that aided identification of WRA and none such exist. The second aim was to identify existing BCIs for identifying any other chronic diseases that could be adapted for use in WRA. Owing to the specificity of context and construct for BCIs in other disease areas, these would not be simply transferable. However, there are some more general insights into BCI design from a recent systematic review by Mather *et al* ^38^ focused on barriers and enablers of behaviour change by primary care HCPs. The authors reported that HCPs commonly perceive those in the ‘capability’ and ‘opportunity’ domains of COM-B, which are linked with interventions related to education, training, restriction, environmental re-structuring and enablement. A specific BCI for use with patients at risk of WRA should be developed using a standardised and established BC methodology, taking account of local variation in employment, industry, and causative exposures.

## Trial registration number

The protocol was registered on the international PROPSERO database of systematic reviews on 19 April 2023 (registration number: CRD42023418316).

## Data

The dataset relied on in this article is available from the corresponding author on reasonable request.
